# odMLtables: A User-Friendly Approach for Managing Metadata of Neurophysiological Experiments

**DOI:** 10.3389/fninf.2019.00062

**Published:** 2019-09-27

**Authors:** Julia Sprenger, Lyuba Zehl, Jana Pick, Michael Sonntag, Jan Grewe, Thomas Wachtler, Sonja Grün, Michael Denker

**Affiliations:** ^1^Institute of Neuroscience and Medicine (INM-6) and Institute for Advanced Simulation (IAS-6) and JARA Institute Brain Structure-Function Relationships (INM-10), Jülich Research Centre, Jülich, Germany; ^2^Theoretical Systems Neurobiology, RWTH Aachen University, Aachen, Germany; ^3^Institute of Neuroscience and Medicine (INM-1), Jülich Research Centre, Jülich, Germany; ^4^Molecular and Systemic Neurophysiology, Department of Neurophysiology, Institute of Biology II, RWTH Aachen University, Aachen, Germany; ^5^Department of Biology II, Ludwig-Maximilians-Universität München, Martinsried, Germany; ^6^Institut for Neurobiology, Abteilung Neuroethologie, Eberhard-Karls-Universität Tübingen, Tübingen, Germany

**Keywords:** metadata management, open metadata Markup Language (odML), reproducibility and tools, graphical user interface (GUI), laboratory routines and automation, electrophysiology

## Abstract

An essential aspect of scientific reproducibility is a coherent and complete acquisition of metadata along with the actual data of an experiment. The high degree of complexity and heterogeneity of neuroscience experiments requires a rigorous management of the associated metadata. The odML framework represents a solution to organize and store complex metadata digitally in a hierarchical format that is both human and machine readable. However, this hierarchical representation of metadata is difficult to handle when metadata entries need to be collected and edited manually during the daily routines of a laboratory. With odMLtables, we present an open-source software solution that enables users to collect, manipulate, visualize, and store metadata in tabular representations (in xls or csv format) by providing functionality to convert these tabular collections to the hierarchically structured metadata format odML, and to either extract or merge subsets of a complex metadata collection. With this, odMLtables bridges the gap between handling metadata in an intuitive way that integrates well with daily lab routines and commonly used software products on the one hand, and the implementation of a complete, well-defined metadata collection for the experiment in a standardized format on the other hand. We demonstrate usage scenarios of the odMLtables tools in common lab routines in the context of metadata acquisition and management, and show how the tool can assist in exploring published datasets that provide metadata in the odML format.

## 1. Introduction

In recent years, the workflows involved in conducting and analyzing neurophysiological experiments have become increasingly complex (e.g., Coles et al., 2008; Denker and Grün, 2016; Brochier et al., 2018). Several factors contribute to this development. Nowadays, a recording setup is usually comprised of several hardware and software components that are often produced by different companies, or might even be custom made. In addition, due to the technological progress in neuroscience during the last decades the task designs have become more and more sophisticated, as can be observed, for example, when considering experiments mimicking realistic, natural conditions. Neuronal or muscular signals (e.g., eye and arm movements) can be gathered in parallel from multiple optical or electrical recording sites (Nicolelis and Ribeiro, 2002; Verkhratsky et al., 2006; Obien et al., 2014) together with complex behavioral measures (Maldonado et al., 2008; Jacob et al., 2010; Vargas-Irwin et al., 2010; Schwarz et al., 2014). Moreover, these signals can be experimentally manipulated in intricate ways, e.g., via multidimensional natural stimuli (Geisler, 2008) or sophisticated optical or electrical stimulation methods (Deisseroth and Schnitzer, 2013; Miyamoto and Murayama, 2015). As a result, the amount of information required to fully describe all circumstances under which the experiment was conducted and data was recorded, here collectively referred to as “metadata”, has grown considerably at the same time. Therefore, metadata of neuroscientific studies are increasingly difficult to document and the implementation of specific software solutions to facilitate their management in daily routines involves a lot of time and effort (Zehl et al., 2016).

The complexity of collecting metadata originates from two factors: Firstly, the growing heterogeneity of setup equipment alone makes it difficult to fully track the exact circumstances under which the primary data were recorded and how the recorded signals were processed along an experimental recording session (“black box” effect, i.e., the difficulty to precisely relate inputs and outputs to the equipment). Secondly, the complexity of the signal types and manipulations using various tools within custom signal processing pipelines increases the effort needed for comprehensive metadata tracking across all parts of the recording system and all processing steps. In particular, the hardware components and software tools employed in these experimental setups typically do not provide a complete account of their metadata and store their output in non-standardized representations that impede gaining insights into the details of the recording process. Nonetheless, collecting and providing metadata of an experiment is a necessary step towards replicable experiments and therefore forms the basis for reproducible research (Tebaykin et al., 2018). In this regard, metadata have to be human readable in order to give users semantic access to the data, similar to a traditional lab book. However, only standardized, machine-readable metadata can be systematically reproduced during automatized analysis processes, which makes them a crucial ingredient for tracking the data provenance leading to a research publication.

A software approach to manage neuroscientific metadata is the *open metadata Markup Language* (odML) framework (Grewe et al., 2011). odML provides a standardized format for organizing metadata of arbitrary type into a hierarchical structure that is both human and machine-readable. With this, it is possible to organize metadata originating from heterogeneous sources in a unified way and record them in an common, interoperable format. Providing metadata in such a standardized format along with the data files of an experiment facilitates the collaboration process between members of a scientific project, because metadata can be organized and made available to all members in a unified way, thus supporting rigor and reproducibility of data analysis through standardized and formalized access to the available metadata (Zehl et al., 2016). The reference implementation of the odML format is based on the generic eXtensible Markup Language (XML). Version 1.4, which is considered here, also supports the JSON and YAML formats and provides an application programming interface (API) for Python and Matlab (http://www.g-node.org/odml).

**Figure 1:**
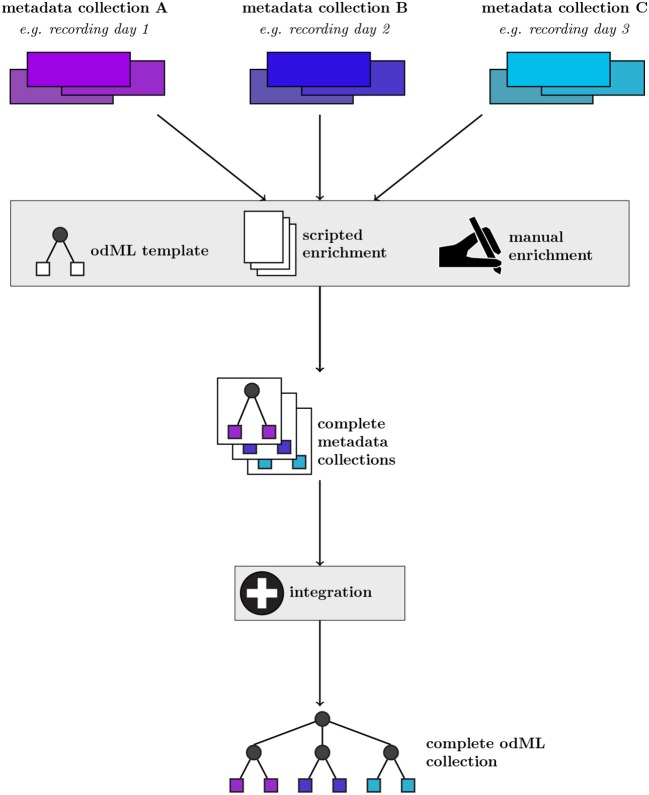
Generic workflow of generating metadata collections from source files using the odML framework. For a given metadata collection (top row, example metadata collections A–C), metadata are pooled from multiple files and enriched via manual entry (second row). These metadata are converted into individual collections via a scripted approach applying an odML template structure (third row). By further integrating individual multiple metadata collections (fourth row), a complete odML collection containing all recordings of a particular experiment can be created (bottom row).

Figure 1 shows a generic representation of an example workflow that results in the generation of a metadata collection represented in odML. The starting point are collections of files containing various subsets of the metadata for individual recordings of an experiment (e.g., different recording days). The data in these files are often organized in different formats within a collection, and files and metadata between different collections may differ due to factors, such as changes in the experiment. Therefore, it is possible and advisable to construct template structures for the metadata collection to enforce a systematic metadata structuring. However, in practical scenarios often custom scripts are required to populate these templates, e.g., to cover small variations between metadata collections when a certain piece of information is not present for a particular recording. In addition, the metadata collection must be manually enriched by information that is not digitally available in the first place. The outcome of this build process are odML files for each recording, adhering to a uniform template structure. In a final step, these individual metadata collections may be merged into a single odML file in order to provide scientists with the ability to perform full metadata queries on the complete experiment. Zehl et al. (2016) provides a complete account of this workflow including practical examples.

Implementing and applying such a rigorous workflow as described in Figure 1 requires programming skills by the scientist. However, metadata handling is often performed by several experimenters with varying computational expertise. Furthermore, extensive manual editing of the metadata files via the present graphical user interface (GUI) included in the odML framework tends to be cumbersome for large metadata trees due to their hierarchical, complex organization. While visualization of the hierarchical organization is suited for an overview of the general structure and relation of the metadata, finding or comparing particular values can be difficult if they are distributed in different branches of the hierarchy. Furthermore, editing of distributed entries is laborious, because a hierarchical organization also requires navigation through the tree to access a particular entry. This makes this metadata management tool inefficient to use in an experimental laboratory where often (i) single particular entries need to be modified manually as the experiment progresses, (ii) a batch of similar entries need to be modified coherently as the data processing progresses. The combinations of all these factors results in many experimental laboratories frequently collecting metadata in flat tabular formats independent of an explicit, underlying hierarchical structure, using tools for generation and manipulation of tables that do not require programming expertise, are widely adopted, readily available and familiar to the experimenters.

Thus, for these purposes a flat tabular representation of the metadata appears to be suitable. It has the advantage of providing easier access to, and simpler visualization of, the metadata than a hierarchical format. Tabular representations of hierarchical structures are implemented in a number of generic software tools for xml representation^1^. However, these generic xml editors do not provide support for using xml to handle scientific metadata in a concise way.

We developed odMLtables as a Python package to complement the odML framework in simplifying working with, and in particular manually editing, the metadata stored in the hierarchical odML format. odMLtables facilitates the integration of the odML framework into the experimental workflow by converting between hierarchical odML and tabular representations in xls or csv format. As opposed to working on the hierarchical odML structure, these tabular formats are easily accessible via familiar spreadsheet tools (e.g., *Microsoft Excel, LibreOffice Calc*) that enable neuroscientists to manually extend or edit the content of an odML metadata file. Vice versa, the ability to convert configurable tabular representations of metadata to odML will help into a robust, self-consistent, and validated format, ready for automation tasks, such as batch analysis processing or integration into databases. Thus, odMLtables acts as a bridge between users and formal representation. In addition, the odMLtables package comprises a GUI that guides the user through all functional features of the tool. Besides the conversion between hierarchical and tabular formats, these features include operations identified as useful in the metadata acquisition process, such as merging or filtering metadata. Implementing these operations would require custom programming efforts in the absence of odMLtables, either by manipulation of odML files using the odML API, or by using programmatic capabilities of the tabular editor. odMLtables also opens access to the odML framework for scientists with little or no programming experience. All main functionality to interact with metadata files is directly accessible from the odML GUI since version 1.4.0. The software has benefited from the experiences gained in applying it in collaborative projects involving three different experiments collecting electrophysiological data: (i) cortical activity in macaque performing a visually-guided motor task (e.g., Brochier et al., 2018; Denker et al., 2018), (ii) cortical and hippocampal activity in a developmental study in mice (e.g., Bitzenhofer et al., 2017), and (iii) cortical activity in a category learning task in gerbils (e.g., Ohl et al., 2001).

The embedding of odMLtables in a real-world metadata management workflow is described in Zehl et al. (2016), resulting in a published dataset with detailed metadata descriptions in the odML format (Brochier et al., 2018). In these publications the focus was on the concepts of metadata management and the detailed experiment description. Here we complement these studies by a technical tool for convenient metadata capture. Because the complexities of the real experiment (an instructed, delayed reach to grasp task with multielectrode recordings from monkey motor cortex) would distract from the presentation of the features and usage of odMLtables, the examples presented here are abstracted from these studies.

To demonstrate the application of odMLtables we present seven minimalistic scenarios of practical metadata management using odML and odMLtables. Together these scenarios form a complete metadata workflow based on an exemplary multi-day experiment as commonly encountered in neurophysiology (cf. Figure 1). However, such scenarios also occur in other fields of science where data is aggregated in repetitive acquisition cycles (e.g., multiple days of measurements). Moreover, the scenarios are of sufficiently generic nature to translate to other situations where metadata information is collected. The first two scenarios demonstrate the first steps for setting up a new metadata workflow and daily metadata collection. Four scenarios deal with the ongoing metadata validation, enrichment and visualization. The last scenario introduces automation of metadata collection and management using odML and odMLtables.

Using these scenarios we demonstrate how odMLtables facilitates access to sophisticated metadata management software odML for non-programmers and with that optimizes routine manual metadata acquisitions in any laboratory workflow. In addition, odMLtables can be used to create visually enhanced tabular overviews of complete or filtered metadata from any hierarchically structured odML files. For a scripted metadata approach a Python interface also permits programmers to benefit from odMLtables features.

## 2. Software Description

**Figure 2:**
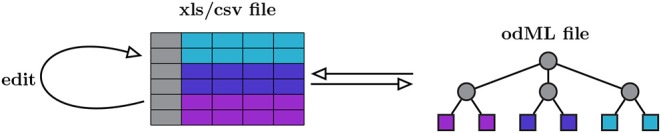
Minimal workflow for manually editing odML files via odMLtables. Metadata is manually edited in tabular form using spreadsheet software and stored in xls and csv formats (left). The minimal functionality of odMLtables is to convert between such tabular representations and the hierarchical odML structure (right). The hierarchical placement of individual metadata entries, i.e., the Sections of the odML tree, is encoded in a specific column of the table (gray boxes and circles), whereas the values and attributes of metadata entries, i.e., a Property represented as leaves of the odML tree, are stored in rows of the table (colored boxes).

odMLtables is a Python package that provides a set of functions for working with metadata descriptions in the odML metadata framework, with a particular focus on making these metadata easily accessible for users. The key approach is to bring the typically complex, hierarchical structure of the odML format into a tabular and reduced representation, such that metadata can be more easily inspected or edited. Therefore, at its core, odMLtables provides functions to convert between the odML format and the corresponding tabular representation which can be represented in the *Microsoft Excel* (xls) or the generic comma separated value (csv) format (Figure 2). Metadata converted to these tabular formats are accessible via widely used spreadsheet software (e.g., *Microsoft Excel*^2^ or *LibreOffice Calc*^3^), such that users are able to intuitively view and edit the metadata. After editing, the metadata can be brought back to the standardized, hierarchical form defined by the odML framework (as illustrated in Figure 2).

Next to the functionality of converting between odML and the tabular formats, odMLtables provides four additional capabilities that address common tasks when working with metadata collections:

- filtering (or reduction) of a metadata collection to a subset
- merging of two metadata collections
- generation of a basic odML structure to facilitate the design of a new metadata collection
- creating a tabular overview across multiple metadata entries within a metadata collection

The functionality of odMLtables can be accessed in one of two ways. First, the API of odMLtables complements the original Python odML API (Grewe et al., 2011). As such, odMLtables simplifies the scripting of automated metadata extraction and aggregation tasks in an experiment. Second, odMLtables includes a GUI that enables non-programmers access to the large majority of functionality offered by the library. In this way, odMLtables can aid work with odML-based metadata collections in metadata workflows that do not include scripted processing stages.

In the following, we describe in detail the structure of the hierarchical and tabular metadata representations, the main capabilities of odMLtables illustrated by means of the GUI, and its internal architecture.

### 2.1. Hierarchical and Tabular Representations of Metadata

#### 2.1.1. Hierarchical Metadata in the odML Format

odML^4^ is a versatile hierarchical format for metadata (Grewe et al., 2011) developed by the German Neuroinformatics Node (G-Node). While it was originally designed for electrophysiological metadata, its generic structure makes it also applicable to other scientific contexts.

**Figure 3:**
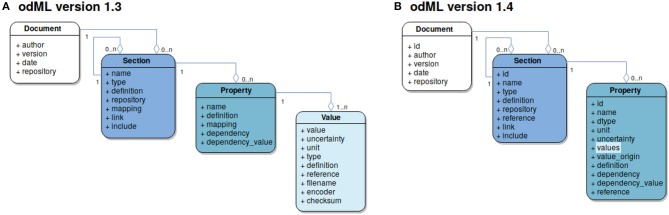
Evolution of the odML data-model. Each box represents an entity defined by the data model and is color coded. Connections between entities are illustrated using the UML aggregation relation where a diamond denotes the target of a connection; the numbers at source and target denote the cardinality of each entity in the connection. **(A)** Version 1.3 data model. Four entities are defined: The *Document* (marked in white) as the root element of a metadata file contains information about the author, document date, the document version and a default repository containing definitions used within the Document. It further contains grouping elements, *Section*s (marked in dark blue). These are defined via their name and type attributes and can hold subsections and provide semantic structure to an odML Document. The “definition” attribute provides information about the nature of a Section, while “link” and “include” refer to further Sections within the same or a different Document, respectively. Sections may contain named *Property* entities (marked in cyan) which hold at least one *Value* (marked in light blue) thus creating an extended key-value pair. **(B)** Version 1.4 data model: To simplify the use of the odML data model the Value entity was integrated into the Property taking over the attributes “dtype” (data type), “unit,” “uncertainty,” “value origin,” and “reference.” In this version a Property may contain a list of values, which must be identical in terms of the relocated attributes thus reducing the risk of ambiguities in the value list. For more information on attributes that have not been modified please refer to the original publication (Grewe et al., 2011). Figure with permission adapted from Grewe et al. (2011).

The basic concept is to use a tree-like structure of **Sections** to store metadata as **Properties** (extended key-value pairs) in a common **Document** (Figure 3B). For example, using this paradigm, parameter settings of a specific device used in the experiment would be represented as Properties collected in a specific Section for that device. For a detailed tutorial^5^ on odML please refer to the online reference documentation^6^. The usage of odML in different environments with varying requirements has led to diversification, the identification of unused features, and the need for improvement of the original data model. In case of the odMLtables project, for example, the original internal data representation required only a subset of the complete odML data model. These and other re-implementations (NIX and RELACS projects) did not fully comply with the original specifications and led to a diversification of the de-facto implemented data models. In order to resolve this situation, with the latest release of odML version 1.4^7^ (i) data model and implemented features were streamlined and adapted to ensure compatibility between the various project implementations and (ii) additional features were introduced. The following paragraph briefly reviews the changes of the data model since its publication in Grewe et al. (2011).

##### 2.1.1.1. odML model revision and streamlining

A number of features were merged or moved by the change from odML version 1.3 to version 1.4 in order to simplify usage of the odML framework as originally described in Grewe et al. (2011), and to mitigate potential ambiguities in the data structure. In the following, we briefly explain two major changes that affected the design and use of odMLtables. The first change was the merging of Value and Property entities (compare Figures 3A and B). This prevents value ambiguities within a Property and reduces the effective file size since the value dependent attributes (“unit,” “uncertainty,” “data type,” and “reference”) are defined only once for a set of values. This change simplified also the tabular representations of lists of values created by odMLtables. Second, for compatibility with the NIX projects' odML implementation, entities now contain a universally unique identifier (UUID, auto-generated identifier with extremely low collision probability) for unique identification of odML entities even across unrelated files to ensure comprehensive provenance tracking, including the ability to create tabular metadata representations across projects using future odMLtables versions. Compatibility for odML files using the old format version is ensured via automatized conversion functionality.

##### 2.1.1.2. Additional features

The odML core library already provides an in-built mechanism to search and retrieve Sections, Properties or values within a Document. The need to consistently search for metadata entities across Documents from different sources led to the development of an export feature of odML metadata to the Resource Description Framework (RDF) format^8^, a general and widely used storage format of graph databases. Multiple odML files exported to RDF can be loaded into any graph database supporting RDF and will be combined into a single graph. Moreover, while XML is still the default storage format, odML now additionally supports storing the metadata in the text based file formats JSON^9^ and YAML^10^. JSON has become a de-facto data exchange standard between web based and standalone computer applications. The support of JSON makes odML metadata more easily consumable in machine-only workflows through modern applications. Since both XML and JSON primarily aim at machine-readability, their structure is not easily readable by humans. To ease reading of raw odML files by actual persons the YAML file format support was added.

For easy visualization and manipulation of specific odML files, the graphical user interface of odMLtables was integrated into the native odML GUI (odml-ui^11^). Thus, the odML GUI now grants direct access to the main odMLtables features, making both software tools even easier to use back to back for both browsing and editing of metadata.

#### 2.1.2. Tabular Representation of the odML Format

**Figure 4:**
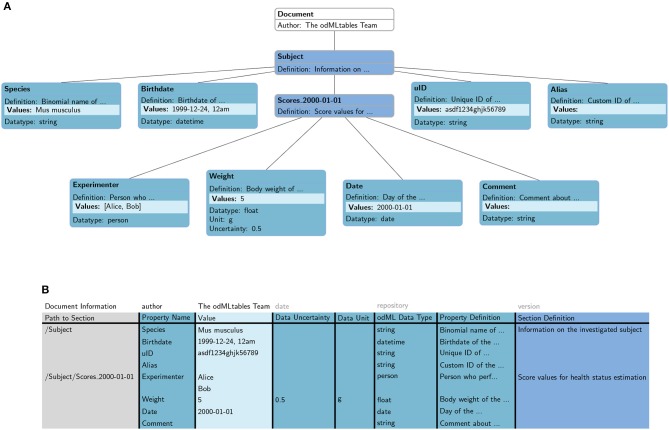
Mapping of an odML structure in **(A)** hierarchical metadata format to **(B)** tabular format. Individual attributes of the odML entities are represented in different columns in the tabular representation (e.g., “Section Definition,” “Property Name,” “Data Uncertainty,” compare color code). Document attributes (“author,” “date,” “repository,” and “version”) are described separately in the first row of the tabular representation. The hierarchy of Sections is captured in an additional column (“Path to Section”) describing the path between the odML Document and the current Section. Each metadata entry in the hierarchical format corresponds to a single row in the tabular format. Items of a list are treated as individual entries.

odMLtables converts the hierarchical odML structure (Figure 4A) into a specific tabular (flat) representation (Figure 4B), stored either in the xls or csv format. In this format, each row corresponds to one particular value entry. The columns further describe the Property and Section each value belongs to, e.g., the Property name, the Section and Subsections the Property belongs to, the physical units, or the Property definition. The hierarchy of Sections in which a Property is located in the original odML structure is represented by a path construct, where individual Section names are delineated by the “/” character. For increased readability, repetitive information (i.e., identical information to the cell above) is optionally displayed only at the first instance (e.g., “Path to Subject” entry (“/Subject”) in row 3, 4, and 5 in Figure 4B). By default, the column headers are predefined (Figure 4B, second row), however the header names can also be customized as long as a mapping between the predefined names and the custom names can be provided. The order of the columns of the table can be customized since the column header names are used to associate columns with attributes of the hierarchical odML structure. The odML Document attributes “author,” “date,” “version,” and “repository” are handled separately and are placed in the top row of the tabular odML representation.

### 2.2. Software Functionalities

odMLtables is a tool that provides five functionalities surrounding work in creating and accessing metadata collections. In the following, we describe the capabilities of these features, while their use is put into the context of a typical workflow in section 3.

**Figure 5:**
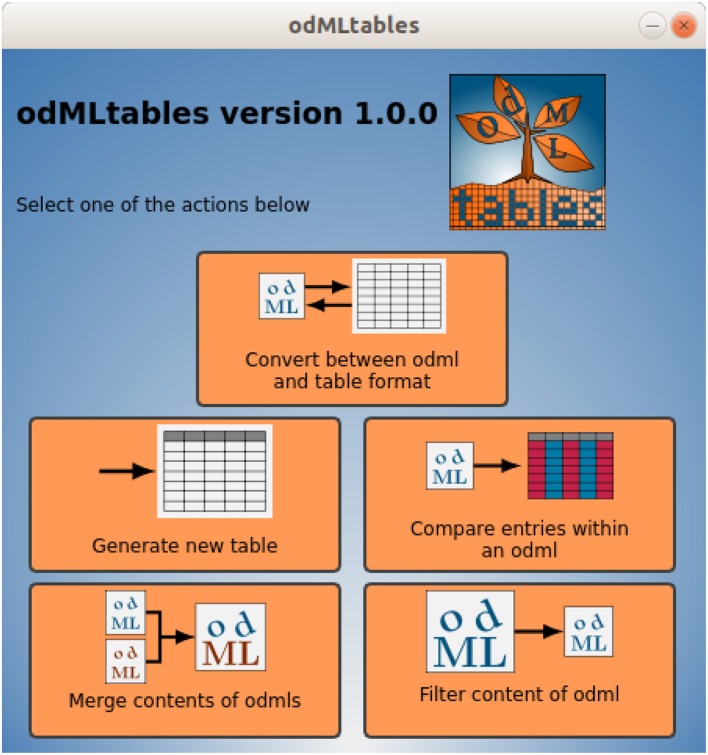
Main window of the odMLtables GUI. The interface gives access to the main functionalities available by the tool: converting files from hierarchical to tabular (flat) representations, generating an empty generic odML table (template), comparing entries within a metadata collection, merging contents of two collections, and selecting a subset of a metadata collection (filtering). Each button starts a series of dialogs (wizards) that guide the user through the corresponding process.

All main features of odMLtables are available via the odMLtables GUI (Figure 5). Upon launching the application, it presents the user with five buttons, each leading to a series of dialogs (wizards) to perform a specific odMLtables functionality. For the more complex dialogs that include a large number of parameters to set, the GUI offers to save and load the dialog configuration to efficiently re-run a functionality with given parameters.

In addition to the functionality offered by the GUI, the Python programming interface of odMLtables offers additional features, most notably, the ability to customize the default values for odML data types. The default values can be displayed using a highlighted coloring scheme to indicate to the researcher that a Property currently contains a default value (for details, see the odMLtables documentation^12^).

The main features of odMLtables are described in detail below and are referred to as features F1–F5:

**F1: Convert between odML and table format**. This function converts metadata collections between the representations in the different file formats odml, xls, and csv. For the conversion to and from the tabular formats (xls/csv) a specific formatting of the table is required in order to interpret the table as hierarchical odML structure (see section 2.1.2). Nevertheless, odMLtables allows for a certain degree of flexibility in order to give researchers the ability to design tabular formats to best fit their workflow. In particular, this encompasses the inclusion or removal of certain optional columns, the arrangement of columns, column headers, or the coloring scheme. Note however, that for the reverse conversion from a tabular format back to the odML format, these customizations need to be known (e.g., custom column names, see section 3.1).
**F2: Generate new metadata collection table**. This function generates and saves an empty, generic (template) odML structure in the xls format. This generic structure provides a good starting point to design a metadata collection or template structure in a tabular format providing the required tabular structure for conversion to a hierarchical odML structure. Similar formatting options can be applied to the table as indicated above.
**F3: Generate overview across entries within a metadata collection**. This function creates a chart listing multiple entries within a single metadata collection. It is intended to develop overview sheets containing similar Properties, e.g., the animal weight at different ages. The generated table does not follow the tabular odML format and can therefore only be used for visualization and not for conversion into the hierarchical odML format. Using common spreadsheet software the comparison table can be saved as a figure and printed for usage in a laboratory notebook.
**F4: Merge contents of two metadata collections**. This function allows to merge multiple files (odML format) into a single file. Here, by default, Sections, Properties and values are added to existing entities during merging. However, for values of coinciding Properties the option exists to overwrite values during the process of merging.
**F5: Filter content of a metadata collection**. This function reduces the size of an odML file based on a filter mechanism, which can include multiple steps of filtering and custom filter functions to select only specific parts of an odML structure. The filter mechanism e.g., can extract all Properties containing no values to present the experimenter potential missing entries in the metadata collection.

### 2.3. Software Architecture

In the following, we explain the internal structure of the odMLtables software. For a detailed description, see the function reference in the odMLtables documentation.

The core of odMLtables is the OdmlTable class, which provides the main functionality for loading and saving metadata collections in the different file formats. It implements basic operations on the loaded metadata independent of the file format they originate from. Within the class, metadata are internally represented as a list of dictionaries, where each dictionary corresponds to an odML Property. Functions that modify the metadata collection, like merging and filtering, act directly on this internal dictionary representation. The two tabular formats xls and csv require additional information regarding the table layout when being saved to disk, e.g., the color scheme. Therefore, two subclasses of the OdmlTable class (OdmlXlsTable and OdmlCsvTable) carry these additional output settings. Finally, a separate CompareSectionTable class implements the function for comparing Properties within one odML structure. As for the OdmlTable class, two specific subclasses for xls and csv output are defined to capture layout information (CompareSectionXlsTable and CompareSectionCsvTable).

One feature of odMLtables in generating xls files is to highlight a value entry if it corresponds to the default value of the corresponding Property's data type. However, the odML library itself does not specify such default values for all of its data types. Moreover, it is not mandatory, nor always desired, to specify a data type in the odML in all circumstances, e.g., when leaving a value empty. Therefore, odMLtables provides functionality to work with default values for data types in the OdmlDtypes class. It manages the data types, synonyms, default values, and value conversions. The class is used for entering default entries when loading empty values from a tabular representation, and for default value highlighting.

In addition to the core module, odMLtables provides a GUI that exposes most functionality of the core module. The GUI is based on the PyQt5^13^ framework and consists of a main window (Figure 5) and five wizards (see section 2.2). Each wizard inherits from the OdmltablesWizard class, which provides helper functions and error handling. The Settings class stores the current user settings for calls of odMLtables core functions, and provides functionality to save and restore user settings between different executions of the GUI.

## 3. Embedding odMLtables in Data Acquisition and Analysis

While most scientists would agree that accurate records of the minute details of an experiment are the foundation of good scientific practice, in the everyday routine of an experimental electrophysiology lab it is difficult for the scientist to record, sort, and maintain the wealth of metadata information that accumulates during an experiment. While the odML format is suitable for storing metadata information from different sources, lacking to date is a set of tools that allows the scientist to create, manipulate and visualize the data stored in this format. In the following, we present commonly encountered scenarios involving metadata handling that originate from our collaborative work. These scenarios touch the issues of how to design the hierarchical structure to store and organize the metadata, how to practically enter metadata before, during or after the experiment, and how to create a comparison of rich metadata buried within the odML structure. It turns out that for each of these scenarios a flattened tabular representation of the metadata is a practical solution that feels intuitive to the user. In the following we demonstrate how to implement these scenarios that consist of combining operations in odMLtables and a spreadsheet program. All scenarios are also available as an interactive Jupyter Notebook^14^ accessible via the odMLtables documentation^15^, and available in pre-executed form as Data Sheet 1.

### Scenario 1: How to Generate a Metadata Template Without Programming

In conducting animal experiments, a typical scenario where metadata are collected manually on a daily basis is the creation of an animal *score sheet*. Such score sheets record quantitative, and in part also qualitative, measures that are collected in order to document and judge the animal's health and state over the duration of the experiment. Often, these sheets are an obligatory piece of documentation of the experiment, such that only the availability of a defined workflow to create score sheets guarantees their consistency over multiple years and different experimenters. For example, for mouse experiments, typical measures are the body weight, water intake and breathing frequency, many of which can be used to assess the health of an animal, e.g., by calculating a health score for each mouse (Foltz and Ullman-Cullere, 1999; Burkholder et al., 2012). In Figure 4 we depicted how metadata of a single, minimized score sheet can be integrated into an odML document containing collective information on a subject.

The measurements for such score sheets are typically easy to perform, and for this reason may be conducted by a number of different people in the lab. Therefore, the daily process must be simple, intuitive, and robust in order to be conducted by all members of the group. Collecting the information in a table format using common spreadsheet software tools, such as *Microsoft Excel* or *LibreOffice Calc*, satisfies these requirements.

**Figure 6:**
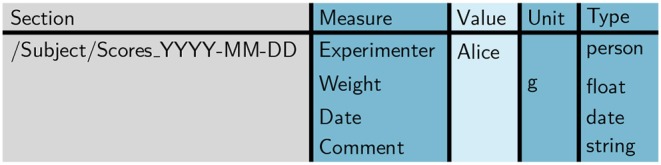
Template score sheet. The template score sheet contains the measures required for each measurement day, including optional default values (here: “Alice” for “Experimenter” and “g” as unit for “Weight”).

To guarantee a consistent structure of such a score sheet, initially a template needs to be set up, i.e., a table containing the measures that are to be recorded on a single day. In order to accomplish this, as a first step we generate an empty template table using odMLtables. To improve the readability, we enter custom column names in odMLtables to create the table (“Section” instead of “Path to Section,” “Measure” instead of “Property Name,” “Unit” instead of “Data Unit,” and “Type” instead of “odML Data Type”). Also we omit the attributes “Section Definition,” “Property Definition,” and “Data Uncertainty” in the context of these example scenarios. As second step, using a spreadsheet, we design the metadata structure for a single score sheet as shown in Figure 6. The value field for each entry can be either left empty or a default value can be entered. The latter case is interesting for values that are likely to be constant for the majority of experiments, e.g., the name of the experimenter. Since the colors of a table saved in the xls format are ignored when converting to the odML format, it is possible to use arbitrary color coding within the spreadsheet software to improve the readability of the table for the experimenters entering the values.

We designed the template table such that it matches the properties of the minimized score sheet section already depicted in Figure 4. Notice that in the template the entry for column “Section” already includes a parent section to reference the animal (cf., Figure 4). This is convenient for defining the position of each score sheet in the odML hierarchy to simplify a later merging process (cf., scenario 2).

### Scenario 2: Collecting Daily Observations in a Common odML Structure

Once the template from scenario 1 is complete, it is copied to a new file on each measurement day, and the copy is filled out by the person taking the measurements. To avoid that metadata are spread across multiple files and potentially multiple locations, we aim to gather the data from multiple days into a single odML file. To achieve this, we use odMLtables to first convert the individual xls file containing an individual score sheet into the odML format, and to subsequently merge these into a common odML structure spanning multiple recording days.

Specifically, for the conversion from the xls to the odML format we use the odMLtables feature F1 (for details of odML features F1–F5, see section 2.2). After the conversion, the current score sheet present in odML format is merged into the common odML document collecting the complete information of an animal using feature F4 on a daily basis. This extends the odML structure of the subject document by an additional Section each recording day. Note that this is possible because the first column of individual score sheets (Figure 6) not only provides a unique Section name for each score sheet, but also indicates the location of the odML Section in the hierarchical structure of the subject document, (e.g., “Subject/Scores_2000-01-01”). The result is a single odML file containing measures collected on all recording days while the source files generated each day can be archived.

The metadata collection containing the merged score sheets of 2 recording days might look like the following: 
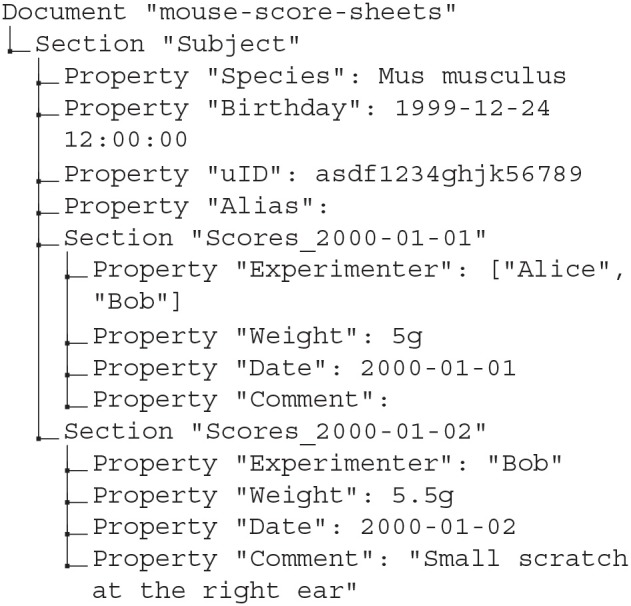


### Scenario 3: Create a Tabular Representation of the odML File for Better Viewing Using the Color Options

**Figure 7:**
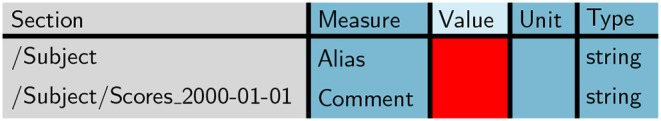
Metadata collection filtered to show only Properties with an empty value. Missing values entries are highlighted in red by odMLtables.

Once the recordings for a number of animals were performed and the corresponding metadata collection is completed, data and metadata should be shared among collaborators in a common repository. In order to get an overview of the data obtained across different animals, the metadata of each animal can be converted into the xls format to simplify the inspection of the associated metadata using spreadsheet software (cf., also Figure 4B). Here, odMLtables provides the option to use color coding and highlighting of default/missing values to improve the readability (Figure 7).

### Scenario 4: How to Filter a Subset of an odML File to Edit It Later on

As the common odML structure grows day by day it is of advantage to extract specific subsets of odML values of interest for visualization using the tabular format. Instead of visualizing the whole metadata collection to periodically verify that all Properties are filled with a value, we can extract a subset of the collection and visualize only the relevant (e.g., empty fields) entries. For this, we use odMLtables feature F5 which can be used to generate an odML that contains only Properties without value information specified. We then convert this reduced odML into a tabular xls representation using odMLtables feature F1. The generated table, as shown in Figure 7 indicating the two empty properties in the odML structure of scenario 2, can be visualized using spreadsheet software and, in case of values not being filled, these can be directly edited manually.

### Scenario 5: Merging the Edited Subset Back Into the Original Structure

The enriched xls sheet generated in step 4 should now be merged back into the common odML structure. For this, we convert it back into the odML format and use the odMLtables merge feature F4 to replace the edited values in the common odML structure with the edited ones. Here, odMLtables merges the two odML files by extending the odML structure and appending metadata entries when the same Property is present in both files. However, when modifying already existing metadata entries in the filtered version this would result in duplication of entries. Therefore, odMLtables offers the possibility to overwrite already existing metadata entries when merging two odML structures. Note that a selective merge of a subset of metadata can be achieved by first filtering the file to be merged using feature F5.

### Scenario 6: Compare Entries in the odML File for Data Screening and Lab Book Usage

In addition to the complete metadata representation as presented in scenario 3, it is possible to generate a reduced overview table containing only plain values of selected Properties. This feature can be used to create a tabular display of Properties of interest (e.g., weight of a specific animal, experimenter who performed the experiment and comments regarding the measurement) in rows for the individual recordings (days) in columns. An example of such a table is given below:

**Table:** 

	Scores_2000-01-01	Scores_2000-01-02
Date	2000-01-01	2000-01-02
Weight	5.0g	5.5g
Experimenter	Alice, ...	Bob
Comment	Blood sample was taken [...]	Small scratch at the right ear

This type of overview tables can also be printed and used as part of the mandatory documentation of the experiment in a written or printed lab book. This way, the recorded data only need to be documented once in a digital fashion and consistency between documentation and digitally available metadata is guaranteed.

### Scenario 7: Automatized Processing of Metadata Collections

After completion of an experiment covering many recording days, the processing steps presented in scenarios 1-6 can be performed in an automatized fashion on the complete metadata collection to generate a comprehensive metadata document and corresponding overviews. While it is possible to perform this action using the graphical user interface, an automated approach has the advantage that it can be repeatedly executed when one of the original files changes, e.g., by a retrospective update of metadata or loss of the generated metadata files. In addition an automated approach is more robust against errors introduced by the manual operation and can be at least partially reused for subsequent experiments.

By use of the odML library together with the odMLtables Python API, users have a rich collection of functions to manipulate and convert metadata stored in the odML format. In this specific example, we show an example script in Listing 1 that loads all daily animal score sheets, adds them to a common metadata structure and exports the final document into an overview and comparative xls sheet for visualization. The code demonstrates the metadata handling workflow by structuring it into a sequence of three generic functions, which can be of use in creating related workflows for different projects.

**Listing 1:**
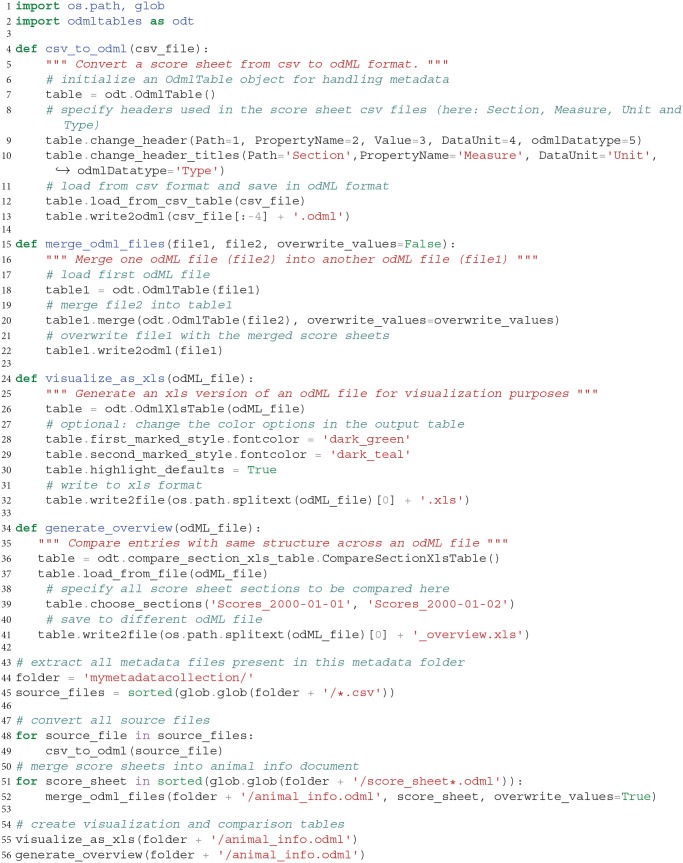
Program to assemble a target odML document covering metadata of multiple recording days by pooling information from multiple csv files and generate visualizations and overviews. Individual functions are automatizing functionalities presented in previous scenarios.

### Improved Handling and Visualization of Complex Metadata Structures

Up to now we demonstrated the basic mechanisms of odMLtables based on highly simplified examples presented above. In a real-world example, however, metadata collections are inherently complex and corresponding metadata collections can easily encompass thousands of values. A publicly available example of this are electrophysiological recordings of macaque monkeys performing a reach to grasp task that include a rich metadata collection stored in the original odML files as well as the corresponding xls representation created by odMLtables (Brochier et al., 2018). We demonstrate the usage of odMLtables to select and visualize a subset of the complete metadata collection as well as generation of overview tables in an interactive Jupyter Notebook in the odMLtables documentation^16^ (available in pre-executed form as Data Sheet 2) as well as in a video tutorial (Supplementary Video 1).

## 4. Discussion

We presented the odMLtables software, which facilitates the use of the odML metadata format in everyday experimental and data analysis work. To illustrate the application of odMLtables in real-world situations, we presented the features of odMLtables in seven scenarios describing a simplified realistic example, namely the definition of an animal score sheet and its use for controlled routine collection of metadata. More specifically, we showed in scenario 1 the setup of a template for an animal score sheet in the csv format and its conversion to odML (F1, F2). In the next scenario, we used this template to routinely collect the animal's health measures and aggregated them in a single odML file per animal (F1, F4). Besides a simplification of metadata acquisition in the csv format, we showed in scenario 3 the benefits of a colored tabular representation for visual inspection of the collected score sheets (F1). In scenario 4, we demonstrated how supplements of metadata values can be easily added by extracting the missing metadata entries from the complete collection (F5). Subsequently we demonstrated in scenario 5 the integration of the amended metadata back into the complete collection (F4). We generated a compressed overview table, summarizing the metadata from different routine collections in a concise format suitable for laboratory notebook in scenario 6 (F3). Finally, in scenario 7 we discussed the automation of the workflow presented in the previous scenarios and provided code examples showcasing the odMLtables Python interface.

**Figure 8:**
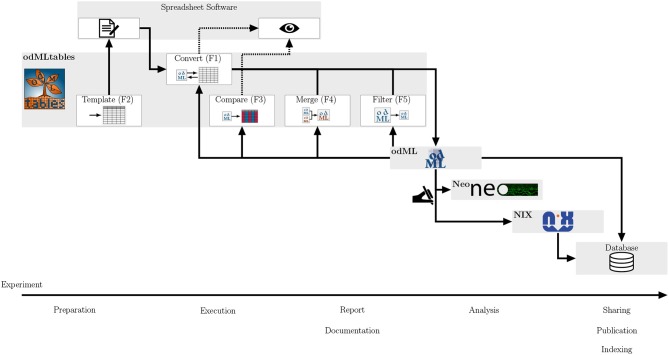
Integrating odMLtables and other software tools in the different stages of an experiment from preparation to publication. During the preparation of an experiment odMLtables in combination with spreadsheet software is used to develop an experiment specific structure of the metadata collection (templates, F2). During the execution and documentation of the experiment, odMLtables converts (F1) between the tabular and odML representations. The compare functionality (F3) is used to generate overviews of odML Properties across different Sections of a metadata collection. The filter (F5) and merge (F4) functionalities are used to create and merge subsets of odML collections, respectively. For analysis and sharing, data can be represented using the Neo framework and annotated with metadata from the odML metadata collection using custom scripts. This combined representation can be saved in a single format using the NIX framework, e.g., to share of data and metadata via a database. In parallel, metadata collections can be incorporated in databases, for example using an export of the odML to the RDF standard.

As odMLtables can be used by programmers and non-programmers alike, it simplifies the development of comprehensive metadata management in the scientific community by offering user-friendly interaction with the odML format. In this way, its usage is intended to improve reproducibility and replicability of experiments and to facilitate cooperative work, both within labs and across different laboratories. Complementing the model scenarios above, in Figure 8 we summarize and generalize the use of individual components of odMLtables during the course of an entire experiment. Although the minimalistic workflow presented here as well as the real-world workflow described in Zehl et al. (2016) and Brochier et al. (2018) are all set in the field of animal experiments covering multiple days, odML as well as odMLtables are not specific to neuroscience and can therefore be used for metadata management in other scientific disciplines. In virtually any experimental research, odMLtables provides benefits on multiple stages of the experiment: from setting up a specific metadata structure in the preparatory phase, manual enrichment of the metadata collection during the experiment, to the generation of overviews and summaries from metadata collections during data analysis. Also, for publicly available datasets with an odML metadata collection, odMLtables can be used to create a tabular representation of the odML files to quickly scan the metadata of the experiment. For example, considering the files comprising datasets hosted on public repositories, tabular, yet arbitrarily formatted representations are commonly used to supply additional information describing the dataset. This information must be parsed by custom codes in order to make it available in the analysis process. In contrast, using odMLtables, such metadata could be transformed into a structured, machine-readable representation with only moderate restrictions on formatting of the xls or csv files. Although odML and odMLtables can be used in a broader context, in the following we discuss specifically its embedding into a tools landscape developing in the field of electrophysiology.

### 4.1. Performance Estimation

Since the release of the original version, odML has been used in various projects for storing metadata as they become available during data acquisition or analysis (e.g., in the NIX^17^ and RELACS^18^ projects), as metadata schema in the EEGbase database^19^ (see also Mouček et al., 2014), and as a part of the metadata data pipeline as described by Zehl et al. (2016) and Brochier et al. (2018). The advantage gained by comprehensive metadata management using odML can be demonstrated by a small example based on a published dataset (Brochier et al., 2018) for which detailed metadata are stored in the odML format. Accessing information about the number of neurons recorded on different electrodes contained in the odML files using common desktop hardware requires ~0.5 s for this dataset using the odML iteration and filter mechanism. Extracting the same information not from the odML metadata but from the original data files using the Python library Neo version 0.7.1^20^ requires about 25 s using the Neo filter and annotation mechanism. Comparing these times, the usage of odML in this example gives a speedup of a factor 50. However, for a fair comparison also the time for odML generation needs to be taken into account, where for a dataset of this complexity a realistic upper bound is on the order of 10 min, considering that the generation process needs to read the data files and a number of associated files (Zehl et al., 2016), and perform various quality or automated preprocessing checks. Comparing this conservative estimate of the generation time of the odML file, the access time using the odML format and the access time using the original data files shows that using the odML format pays off after 25 times of metadata access. This is a relatively small number of metadata accesses for a single dataset considering the relevance of metadata in multiple steps of the experiment, e.g., exploratory analysis and parameter scans in analyses runs, and collaborative work, where different people access the same metadata on different computers. In the latter setting using odML is also of advantage because the potentially large original data files might not be present on all computers of all collaborators, whereas odML files are much smaller in file size and can therefore be shared more easily, e.g., via a version control system like git^21^.

### 4.2. odMLtables as Conversion Tool

One may argue that extending the existing odML editor to support a flattened view on the metadata is a more direct and efficient way to implement tabular representations, as opposed to a converter (such as odMLtables) between formats. However, such a solution has direct implications on (i) the maintainability of the tool, (ii) its adoption by the community, and (iii) its interoperability in the heterogeneous types of workflows typically encountered in data acquisition. Regarding (i), the development of graphical editors for tabular data is a time-consuming endeavor and leads to a complex code base that is difficult to maintain. This is even more true in a scientific environment, where software maintenance is often left to persons who are not expert in GUI programming and design patterns for graphical applications. Regarding (ii), spreadsheet software is already commonly used in laboratory environments to track metadata, and experimental scientists are used to efficiently use these tools in their daily routine. Therefore, integrating such software in a digitized workflow, rather than proposing an entirely new user-facing tool, is bound to lower the threshold for adoption in a laboratory. Finally, regarding (iii), data acquisition workflows in an experimental environment are often subject to constraints set by the individual formats in which metadata are generated by the components of the experimental setup. Tabular representations, and in particular those stored in the csv format, represent *per-se* one of the most commonly encountered and most simple formats to exchange data. Indeed, the capability to read csv data files is provided by the standard libraries of many programming languages, in particular those commonly used in data analysis and scientific computing, such as Python, Matlab, or R. Therefore, being able to convert between human readable tabular metadata generated automatically by various metadata sources of the experiment and their joint representation in a hierarchical odML metadata collection is helpful in creating a metadata acquisition workflow that is interoperable with the various components of the experiment. Combining such workflows with version control systems, such as git, to store the hierarchical or tabular metadata representations is a viable option to enable collaborative creation of metadata records, in particular when considering the text-based csv or odML formats.

### 4.3. Relation to Electronic Laboratory Notebooks

One particular case where flexible interoperability is in demand are electronic laboratory notebooks (ELNs) which are available from a large range of manufacturers and are becoming increasingly utilized by laboratories (Kwok and Kanza, 2018). Their design is actively being researched in the process of digitizing the research process (Kanza et al., 2017). ELNs are software tools originally designed to replace the hand-written lab book used in experimental sciences to document experiments, outcomes and analyses by providing a method to electronically enter such metadata in a digitally signed and potentially encrypted fashion that ensures protection from falsification. Some ELNs go beyond this functionality by integrating tightly with laboratory inventory management systems (LIMS) or analysis pipelines [comparisons of selected ELNs can be found in Rubacha et al. (2011) and various web resources^22^, ^23^, ^24^]. One major advantage of ELNs that store hard metadata (Grewe et al., 2011) in form of key-value pairs is that they can be directly digitally accessed in analysis scripts, rather than having to manually copy the information from the hand-written lab book (Zehl et al., 2016). While for some disciplines specialized lab notebook software packages have been developed (Kwok and Kanza, 2018) that are aware of community standards for storing such metadata, most of these packages come with their own format for storing data that can only be accessed via file export functionality or specific APIs. In some disciplines this may be of little importance, since either the metadata records stored in the ELN are not required in the analysis process, or the metadata are captured using a domain-specific ELN that is integrated with functionality to directly perform the analysis steps from within the ELN. Nonetheless, other disciplines, such as neurophysiology, require detailed metadata available in an environment suitable for performing complex, exploratory analysis protocols that go beyond the capabilities of currently available ELNs. Here, odML is a potential candidate for implementing such features. In absence of a global standard to record metadata, csv represents one of the de-facto standards to export metadata from ELNs in a universal format. For this reason, the conversion to odML via odMLtables provides access to metadata recorded with ELNs for external analysis pipelines that rely on hierarchically structured metadata collections. The same holds true for the reverse direction, where metadata generated by tools building on the odML specifications can be imported into an ELN. For example, the feature of odMLtables to create tabular overviews of the metadata (feature F3, see section 2.2) would allow to generate current overview tables in terms of animal score sheets as csv that could be directly (and assuming the ELN has an API, even automatically) integrated into the documentation of an experiment contained within an ELN, assuming only basic csv import capabilities.

Beyond ELNs, labs increasingly resort to institution-wide databases to manage and record their research activities, and, in some cases, even the data as such. Depending on the architecture, some systems are likely to implement data imports using tabular schemata. One example of such a tool implementing database and processing functionality is DataJoint^25^ as a tool to assist in ingesting, combining and analyzing heterogeneous data in a relational database (Yatsenko et al., 2015). It is easy to populate a DataJoint database using tabular data, as described in detail in the accompanying online documentation. For example, one may extract a subset of the metadata in form of a comparison table using the odMLtables feature F3, and then incorporate this table into a larger DataJoint database spanning all experiments using a generic function for populating from csv tables. In such a fashion, odMLtables presents a gateway to integrate structured metadata by the diverse tools used in a laboratory to organize the record keeping of an experiment.

### 4.4. Outlook

The current version of odMLtables provides a set of core functions that were identified as necessary in co-designing various data and metadata acquisition workflows in collaboration with multiple laboratories spanning different types of experiments and data modalities. Nevertheless, a number of additional features are envisioned as a result of feedback received from these collaborations to extend the range of applications for the tool and enhance its flexibility for heterogeneous metadata workflows. In addition, feature requests are welcome on the project's issue system on github. One next step will be to extend the capability to create tabular comparisons (feature F3) across metadata stores in multiple files. This would give researchers the option to query for metadata that are distributed over several, even differently structured, odML files. For example, in chronic recordings of brain activity accumulated over the course of multiple months, researchers may decide to generate a single odML file per recording day, and may want to utilize such a functionality to compare the number of trials and other performance measures across the entire recording period.

A second planned feature addition to odMLtables, related to the previous aspect, is the ability to create complete tabular representations (i.e., feature F1) across multiple odML files, and vice versa. To this end, one may implement an additional column next to the odML path and Property name that indicates the file in which a certain metadata entry is found. As an example application, one may consider a complex experiment where metadata originating from different parts of the experiment are stored in separate odML files, but a large overview table is desired for manually browsing the metadata. While this is already possible by merging (F4) individual files and then converting (F1) the table, the information about the origin of metadata in the original file structure is lost.

A third feature addition to odMLtables is the automatic generation of Python code based on the steps the user performs in the graphical user interface. For example, this may yield the Python code to perform a certain filter operation designed in the GUI. This would simplify the automation of metadata processing without specific knowledge about the odMLtables API.

For communicating the structure of a complex metadata collection to new collaborators neither tabular nor hierarchical views have been found to be efficient. For this, a graphical representation of the metadata structure is likely to be more useful, especially for large metadata collections. For this reason, a fourth addition to odMLtables would be to introduce a common graphical representation as new output conversion format.

Lastly, as a fifth feature addition, odMLtables could assist scientists in defining the links between data and metadata in an experiment. Typically, several metadata are accumulated from various sources in an experiment that are directly related to one particular part of the data, and in fact, may be crucial in performing data analysis. For example, the signal recorded from a particular electrode may contain the impedance as measured by the manufacturer as well as noise estimated from a pre-processing step. Due to the heterogeneity of experiments and metadata descriptions, it is currently not feasible to establish these connections between data and metadata automatically, e.g., using a predefined mapping based on Property values. Instead, the mapping is carried out manually by implementing customized code that annotates data with metadata during the loading process. Even when data can be loaded via standardized data framework (e.g., Neo^26^, see also Garcia et al., 2014), the annotation of data objects with metadata taken from a standardized metadata collection (e.g., odML), has to be performed independently (see Figure 8). This complicates the process of reading data, and is not transparent to an external user. A possible way of reducing the implementation effort to create experiment-specific annotation of data with metadata, would be to store the relations between data and metadata directly in the metadata structure. For example, using odMLtables, we suggest to add supplementary fields to the table that directly link blocks of metadata to specific data, e.g., to channels with a certain channel ID or to events with specific IDs. In this way, compact objects containing both data and selected metadata could be loaded using a single, generic loading routine. Moreover, by providing odMLtables with a feature to export to NIX^27^, e.g., using the odML-NIX conversion tool^28^, as an additional output file format that combines odML-like metadata with primary data (Stoewer et al., 2014), such that combined data/metadata objects could be easily serialized to disk.

Validation of user generated input is implemented on the level of odML: When saving or loading an odML file via odMLtables or any other method, the odML structure is checked for basic integrity (e.g., consistency of data types and values). It is intended to support custom, user defined validations in future releases. That is, users will be provided with the means of defining own validations to check for required Sections, Properties or Values and combinations thereof. These additional validations will be directly stored within the odML files. They can be applied to ensure metadata consistency even if the file is handled on a different system or by a different person. For example users would be able to define specific Values as required for a particular Property or make sure a Section tree with an experiment-specific content is present before the file can be saved.

In recent years, the scientific community has begun to recognize the need for developing workflows that enable rigorous data management not only to ensure reproducibility, but also to expedite research through efficient data sharing among scientists. The principles governing corresponding data management practices are summarized under the FAIR (Findable, Accessible, Interoperable, Reusable) principles (Wilkinson et al., 2016). The requirement to make data globally findable has lead to the emergence of multiple resources commonly subsurmised under the term “Knowledge Graph,” referring to a graph-like linkage of metadata through an appropriate ontologies, for example, as done in the Knowledge Graph of the Human Brain Project^29^. The resource description format, RDF^30^, is a semantic web technology that provides one possible standard interface to populate such metadata graphs (cf., Figure 8). The complexity of creating RDF descriptions from scratch can be simplified by exploiting the functionality of odML to export RDF schemata from odML files. In this context, odMLtables can be incorporated as a bridge to support researchers in easily entering predefined metadata schemata to expose their data records in large-scale Knowledge Graph infrastructures.

odMLtables is actively developed and a comprehensive documentation including a tutorial is available for release versions on ReadtheDocs^31^ and the latest version can be obtained from GitHub^32^. Future developments of odMLtables include the ongoing embedding of odMLtables in different neuroscientific data and metadata aggregation workflows, and, as a long term prospect, odMLtables is planned to become a fully integrated component of the odML and NIX libraries.

## 5. Current Code Version

**Table:** 

Code version	1.0.0
Permanent link to code/repository	https://github.com/INM-6/python-odmltables
Documentation	https://odmltables.readthedocs.io
Support	https://github.com/INM-6/python-odmltables/issues
Programming Language	Python
Key Dependencies	odML, PyQt5
Research Resource Identifier (RRID)	SCR_016228
Legal Code License	BSD 3-Clause
Logo	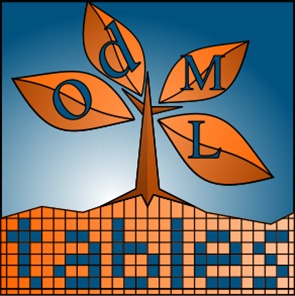

## Data Availability

Publicly available datasets were analyzed in this study. This data can be found at: https://doi.org/10.12751/g-node.f83565.

## Author Contributions

JS and LZ were involved in the design and development of the odMLtables software. JP developed a prototype of the odMLtables software. LZ initiated the odMLtables project. MS, JG, and TW were involved in the development of the odML software. All authors contributed to writing the manuscript.

### Conflict of Interest Statement

The authors declare that the research was conducted in the absence of any commercial or financial relationships that could be construed as a potential conflict of interest.

## Footnotes

^1^See e.g., https://www.oxygenxml.com/xml_editor/xml_grid_editor.html or http://rustemsoft.com/xfox.aspx

^2^https://products.office.com/en-us/excel

^3^https://www.libreoffice.org/discover/calc

^4^https://github.com/G-Node/python-odml, RRID:SCR_001376

^5^https://github.com/G-Node/python-odml/blob/master/doc/tutorial.rst

^6^http://g-node.github.io/python-odml

^7^https://github.com/G-Node/python-odml/releases/tag/v1.4.0

^8^http://www.w3.org/TR/rdf-primer

^9^https://json.org

^10^https://yaml.org

^11^https://github.com/G-Node/odml-ui

^12^https://odmltables.readthedocs.io

^13^https://wiki.python.org/moin/PyQt

^14^https://jupyter.org/

^15^https://odmltables.readthedocs.io/en/latest/tutorial.html

^16^https://odmltables.readthedocs.io/en/latest/tutorial.html

^17^https://github.com/G-Node/nix, RRID:SCR_016196

^18^https://github.com/relacs/relacs

^19^http://eeg2.kiv.zcu.cz:8080/home-page?1, RRID:nif-0000-08190

^20^https://github.com/NeuralEnsemble/python-neo/releases/tag/0.7.1

^21^https://git-scm.com

^22^https://datamanagement.hms.harvard.edu/electronic-lab-notebooks

^23^https://www.labfolder.com/electronic-lab-notebook-eln-research-guide

^24^https://www.gurdon.cam.ac.uk/institute-life/computing/elnguidance

^25^https://datajoint.io/, RRID:SCR_014543

^26^https://github.com/NeuralEnsemble/python-neo, RRID:SCR_000634

^27^http://g-node.github.io/nix, RRID:SCR_016196

^28^https://github.com/G-Node/nix-odML-converter

^29^https://www.humanbrainproject.eu/en/explore-the-brain

^30^https://www.w3.org/RDF

^31^https://odmltables.readthedocs.io/en/latest

^32^https://github.com/INM-6/python-odmltables/

## Supplementary Material

Supplementary Video 1 Video demonstration of the graphical user interface of odMLtables.

Data Sheet 1 Pre-executed Jupyter Notebook implementing scenarios 1 to 7.

Data Sheet 2 Pre-exectuted Jupyter Notebook demonstrating the use of odMLtables with a complex electrophysiology dataset.
