# Supplementary material for: odMLtables: A User-Friendly Approach for Managing Metadata of Neurophysiological Experiments
Source: Front Neuroinform. 2019 Sep 27;13:62. doi: 10.3389/fninf.2019.00062 (PMC6776611; doi:10.3389/fninf.2019.00062)
Supplement: Data Sheet 1 — Pre-executed Jupyter Notebook implementing scenarios 1 to 7. [file Data_Sheet_1.pdf]

demo_scenarios

August 1, 2019

1 odMLtables scenarios

This tutorial is an implementation of the scenarios described in *Sprenger et al (in prep.) odMLtables: A user-friendly approach for managing metadata of neurophysiological experiments* The scenarios present a simple, but realistic use case of odML and odMLtables in an experimental lab and are a good start to start using odML and odMLtables. Modification of this jupyter notebook is highly encuraged and can serve as a starting point for your own metadata workflow. For a detailed description of the individual scenarios, see *Sprenger et al. (in prep)*.

To execute the steps of the tutorial, press *Ctrl* + *Enter* in the cell you want to execute.

1.0.1 Scenario 1: How to generate a metadata template without programming

This scenario describes how a template structure for daily data collection can be set up. The example used here is the measurement of basic attributes of a mouse. The measures collected on a single day can be listed in a table as shown below, where *'YYYY-MM-DD'* specifies the measurement date for score sheet values of a subject.

Section	Measure	Value	Unit	Type
/Subject/Scores_YYYY-MM-DD	Experimenter	Alice		float
	Weight		g	float
	Date			date
	Comment			string

This table can be generated using any spreadsheet software. Possible formats supported by odMLtables are *.xls* and *.csv*. A possible implementation using Microsoft Excel or LibreOffice Calc can include color coding to aid visual inspection and might look like this

from 2019-08-01 16-53-10.bb

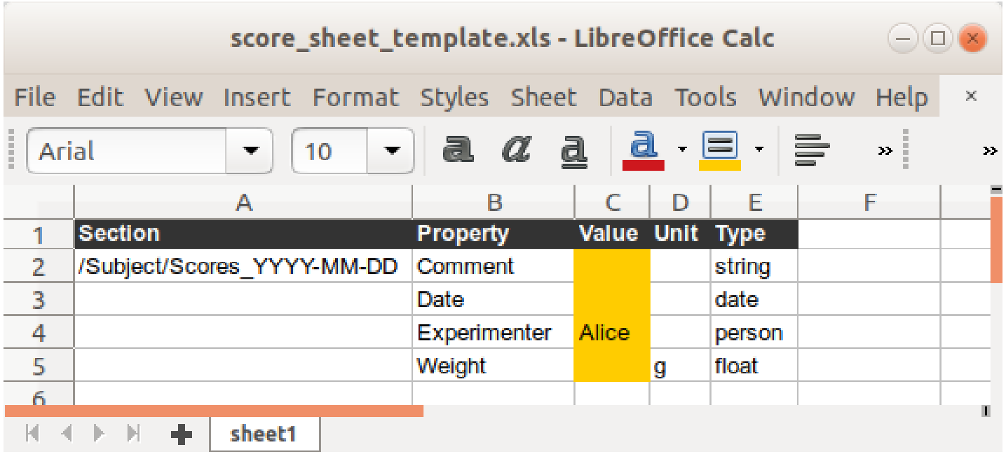

score_sheet_template.xls - LibreOffice Calc

	A	B	C	D	E	F
1	Section	Property	Value	Unit	Type	
2	/Subject/Scores_YYYY-MM-DD	Comment			string	
3		Date			date	
4		Experimenter	Alice		person	
5		Weight		g	float	

Screenshot

For simplicity, we generate a *.csv* file with the same content using Python here.

[ ]:
```
# string representation of the score sheet in csv format
score_sheet_template = \
"""Section,Measure,Value,Unit,Type
/Subject/Scores_YYYY-MM-DD,Experimenter,Alice,,person
,Weight,,g,float
,Date,,,date
,Comment,,,string
"""
# write the string representation to disk
with open('score_sheet_template.csv', 'w+') as f:
    f.write(score_sheet_template)
```

This metadata template in *.csv* format can be converted to an odML file using odMLtables:

[ ]:
```
import odmltables as odt

def csv_to_odml(csv_file):
    """Convert a score sheet from csv to odML format."""

    # initialize an OdmlTable object for handling metadata
    table = odt.OdmlTable()
    # specify experiment specific headers used in the score sheet csv files (Date, Measure, Unit and Type)
    table.change_header(Path=1, PropertyName=2, Value=3, DataUnit=4, odmlDatatype=5)
    table.change_header_titles(Path='Section',PropertyName='Measure', DataUnit='Unit', odmlDatatype='Type')

    # load from csv format and save in odML format
    table.load_from_csv_table(csv_file)
    table.write2odml(csv_file[:-4] + '.odml')
```

```
# convert the score sheet to odml format
csv_to_odml('score_sheet_template.csv')
```

The resulting odML file can be visualized in the browser using the `odml.xls` style sheet. When working locally on your computer, you can generate this visualization by opening the odML file in your browser while having the style sheet located in the same folder.

```
[ ]: # This is utility code for displaying the odML file as html representation here.
# You can also just open the odML file in your browser having the style sheet␣
 ↪in the same location as your odML file and
# will get the same result
from IPython.display import display, HTML
import lxml.etree as ET

def display_odML_as_html(odML_file, xsl_file='odml.xsl'):
    # generate html representation from odML file and style sheet
    dom = ET.parse(odML_file)
    xslt = ET.parse(xsl_file)
    transform = ET.XSLT(xslt)
    newdom = transform(dom)

    # display html
    display(HTML(ET.tostring(newdom, pretty_print=True).decode()))

display_odML_as_html('score_sheet_template.odml')
```

In the same manner as for the daily score sheet, we can also set up a table containing information about the subject. Here, we are covering the 'Species', 'Birthday', a unique subject identifier ('uID') and an alias name gives to the subject ('Alias'). Since this only needs to be filled once for the subject we directly provide the available information for the current subject and don't set up an empty template structure.

Section	Measure	Value	Unit	Type
/Subject	Species	Mus musculus		float
	Birthday	1999-12-24 12:00:00		datetime
	uID	asdf1234ghjk56789		date
	Alias			string

We convert this also into the odML format using the 'csv_to_odml' function and visualize the subject information using the 'display_odML_as_html' function.

```
[ ]: # string representation of the score sheet in csv format
animal_info = \
"""Section,Measure,Value,Unit,Type
/Subject,Species,Mus musculus,,string
,Birthday,1999-12-24 12:00:00,,datetime
,uID,asdf1234ghjk56789,,string
,Alias,,,string
```

```
"""
# write the string representation to disk
with open('animal_info.csv', 'w+') as f:
    f.write(animal_info)

# conversion to odML
csv_to_odml('animal_info.csv')

# visualization using html representation
display_odML_as_html('animal_info.odml')
```

1.0.2 Scenario 2: Collecting daily observations in a common odML structure

The score sheet template structure defined in `scenario 1` can now be copied for each measurement day and filled. The filled files will then be converted to odML and incorporated in a single odML file containing the complete metadata collected for an animal.

Here again we generate a filled metadata sheet in csv format using Python. In a real case this step would be performed using any spreadsheet software.

[ ]:
```
# string representation of the score sheet in csv format
score_sheet = \
"""Section,Measure,Value,Unit,Type
/Subject/Scores_2000-01-01,Experimenter,Alice,,person
,,Bob,,,
,Weight,5,g,float
,Date,2000-01-01,,date
,Comment,,,string
"""
# write the string representation to disk
with open('score_sheet.csv', 'w+') as f:
    f.write(score_sheet)

# convert the score sheet to odml format
csv_to_odml('score_sheet.csv')
```

Now, we have an odML file for the first recording day. To merge these with the information about the subject, we use the `merge` functionality provided by odMLtables and extend the `animal_info.odml`. Since the two odML files have disjoint metadata entries, we don't need to specify the `overwrite_values` parameter of the merge method here.

[ ]:
```
def merge_odml_files(file1, file2, overwrite_values=False):
    """ Merge one odML file (file2) into another odML file (file1)"""
    # load first odML file
    table1 = odt.OdmlTable(file1)
    # merge file2 into table1
    table1.merge(odt.OdmlTable(file2), overwrite_values=overwrite_values)
    # overwrite file1 with the merged score sheets
    table1.write2odml(file1)
```

```
# merge the daily score sheet into the complete metadata collection
merge_odml_files('animal_info.odml',
                 'score_sheet.odml')
```

In the next step we aquire a second set of measurements, recorded on day 2. We directly convert the generated `csv` file into the odML format.

[ ]:
```
# string representation of the score sheet in csv format
score_sheet = \
"""Section,Measure,Value,Unit,Type
/Subject/Scores_2000-01-02,Experimenter,Bob,,person
,Weight,5.5,g,float
,Date,2000-01-02,,date
,Comment,Small scratch at the right ear,,string
"""
# write the string representation to disk
with open('score_sheet.csv', 'w+') as f:
    f.write(score_sheet)
# convert the score sheet to odml format
csv_to_odml('score_sheet.csv')

# merge the daily score sheet into the complete metadata collection
merge_odml_files('animal_info.odml',
                 'score_sheet.odml')
```

Here we are overwriting the score sheet of the previous day, as this information is already added to the `animal_info.odml`.

1.0.3 Scenario 3: Create a tabular representation of the odML for better viewing using the color options

For visualization of the metadata we convert the odML file to the tabular representation in the `.xls` format. This has the advantage of color support within the tabular structure. All color options can be customized using odMLtables.

[ ]:
```
def visualize_as_xls(odML_file):
    """ Generate an xls version of an odML file for visualization purposes """
    table = odt.OdmlXlsTable(odML_file)
    # optional: change the color options in the output table
    table.first_marked_style.fontcolor = 'dark_green'
    table.second_marked_style.fontcolor = 'dark_teal'
    table.highlight_defaults = True
    # write to xls format
    table.write2file('.'.join(odML_file.split('.')[:-1]) + '.xls')

# visualize the complete metadata collection in the xls format
visualize_as_xls('animal_info.odml')
```

1.0.4 Scenario 4: How to filter a subset of an odML to edit it later on

For larger experiments the generated odML structure will grow in complexity. For easier visualization and modification / update of data we will generate an odML file which contains only a subset of the complete score sheet using the odML filter function.

[ ]:
```
def extract_subset(odML_file):
    """Extract comments for the first day in this millenial."""
    table = odt.OdmlTable(odML_file)
    # extract properties, which have no value information
    table.filter(Value=[])
    # generate separate file containing only subset of the information
    table.write2odml('animal_info_filtered.odml')

# extract a subset of the information to a different file
extract_subset('animal_info.odml')
```

The next step could be to convert the filtered odML file into a csv file, update the necessary entries and convert it back into the odML format to finally merge the change back into the complete score sheet. For demonstration purposes here, we will modify the filtered odML file directly and merge it into the complete score sheet.

1.0.5 Scenario 5: Merging the edited subset back into the original structure

[ ]:
```
# this code mimics a manual modification of an existing odML file, e.g. using␣
 ↪the csv representation generated with odMLtables
import odml
odmlfile = odml.fileio.load('animal_info_filtered.odml', show_warnings=False)
odmlfile['Subject'].properties['Alias'].values = ['mouse_134']
odmlfile['Subject']['Scores_2000-01-01'].properties['Comment'].values = ['Blood␣
 ↪sample was taken and shows no abnormalities']
odml.fileio.save(odmlfile, 'animal_info_filtered.odml')
```

For merging the changes back into the complete score sheet we can use the same function as in `scenario 2`. However, in case of editing already existing metadata entries, we need to overwrite outdated entries in the original odML file.

[ ]:
```
# merge the modified filtered odML into the complete metadata collection
merge_odml_files('animal_info.odml', 'animal_info_filtered.odml',␣
 ↪overwrite_values=True)
```

1.0.6 Scenario 6: Compare entries in the odML via data screening, lab book tables

For many odML files a number of metadata structure are repeating within the file. Here, all metadata sections for the daily measurement have the same structure. For visualization and documentation purposes in labbooks an overview across these related structures is usefull and can be generated using the odMLtables `compare` function.

[ ]:
```
def generate_overview(odML_file, sections='all'):
    """ Compare entries with same structure across an odML file """
```

```
    if sections=='all':
        # compare between all available score sheet sections
        sections = [s.name for s in odml.fileio.load(odML_file,␣
 ↪show_warnings=False)['Subject'].sections]
    table = odt.compare_section_xls_table.CompareSectionXlsTable()
    table.load_from_file(odML_file)
    # specify all sections to be compared
    table.choose_sections(*sections)
    # save to different odML file
    table.write2file('.'.join(odML_file.split('.')[:-1]) + '_overview.xls')

# compare all properties across the complete metadata collection
generate_overview('animal_info.odml')
```

This generates an `xls` overview table comparing the first value entries for all selected sections.

1.0.7 Scenario 7: Automatized processing of metadata collections

The workflow presented in `scenario 1 to 6` can be to some extend automatized using odMLtables. This simplifies the generation of an comprehensive metadata collection for the experimenter and makes the workflow more robust against human errors.

Here we start from a collection of daily csv sheets and generate a complete metadata collection as well as overview sheets from these.

In the first step we generation a number of score sheets containing dummy data to demonstrate the metadata workflow building on top of these files.

[ ]:
```
# generate a number of score sheets for demonstration of workflow
import os
import numpy.random as random

def generate_dummy_score_sheets(folder):
    """ Generate 20 daily score sheets with random values entered"""
    # make sure folder exists
    if not os.path.exists(folder):
        os.mkdir(folder)

    # generate score sheets and save them into the folder
    for i in range(20):
        score_sheet = \
"""Section,Measure,Value,Unit,Type
/Subject/Scores_2000-01-{0:02d},Weight,{1:.1f},g,float
,Experimenter,{2},,string
,Date,2000-01-{0:02d},,date
,Comment,{3},,string
""".format(i+1, random.uniform(low=4.0, high=6.5), random.
 ↪choice(['Alice','Bob']),
           random.choice(['','Blood sample shows no abnormalities','Blood␣
 ↪sample shows infection'], p=[0.85,0.1,0.05]))
```

```
        with open(folder + '/score_sheet_day{}.csv'.format(i), 'w+') as f:
            f.write(score_sheet)

def generate_animal_info(folder):
    # string representation of the score sheet in csv format
    animal_info = \
"""Section,Measure,Value,Unit,Type
/Subject,Species,Mus musculus,,string
,Birthday,1999-12-24 12:00:00,,datetime
,uID,asdf1234ghjk56789,,string
,Alias,,,string
"""
    # write the string representation to disk
    with open(folder + '/animal_info.csv', 'w+') as f:
        f.write(animal_info)

# generate multiple daily score sheets for demonstation purposes
generate_dummy_score_sheets('./complete_workflow')
generate_animal_info('./complete_workflow')
```

In the second step we define the complete workflow for metadata collection, merge, storage and visualizations in a single function and run this on the dummy data generated before. An example of one of the dummy data sets is available here.

[ ]:
```
# metadata workflow based on previously generated collection of csv files
import glob
def process_all_metadata(folder):
    """ Find daily score sheets, merge them into complete metadata collection␣
 ↪and generate visualizations. """
    # extract all metadata files present in this folder
    source_files = sorted(glob.glob(folder + '/*.csv'))
    if not source_files:
        return None

    # convert all source files
    for source_file in source_files:
        csv_to_odml(source_file)
    # merge score sheets into animal info document
    for score_sheet in sorted(glob.glob(folder + '/score_sheet*.odml')):
        merge_odml_files(folder + '/animal_info.odml', score_sheet,␣
 ↪overwrite_values=True)

    # create visualization and comparison tables
    visualize_as_xls(folder + '/animal_info.odml')
    generate_overview(folder + '/animal_info.odml')

# run complete metadata workflow from score sheet detection to visualization␣
 ↪generation
```

```
process_all_metadata('./complete_workflow')

# copy style sheet for visualization in browser
os.popen('cp odml.xsl ./complete_workflow/odml.xsl')
```

This generates in addition to the dummy score sheet in the subfolder `complete_workflow` a complete metadata collection in a single odML file as well as two `xls` files for visualization of the odML structure and an overview across all common properties within the complete metadata collection.
