# Supplementary material for: odMLtables: A User-Friendly Approach for Managing Metadata of Neurophysiological Experiments
Source: Front Neuroinform. 2019 Sep 27;13:62. doi: 10.3389/fninf.2019.00062 (PMC6776611; doi:10.3389/fninf.2019.00062)
Supplement: Data Sheet 2 — Pre-exectuted Jupyter Notebook demonstrating the use of odMLtables with a complex electrophysiology dataset. [file Data_Sheet_2.pdf]

demo_complex_experiment

August 1, 2019

1 Handling of experimental metadata using odMLtables

This notebook presents the interaction with metadata from an experiment using odMLtables. Here we use the metadata of a complex electrophysiology experiment published in *Brochier T. et al. Massively parallel recordings in macaque motor cortex during an instructed delayed reach-to-grasp task. Sci. Data 5:180055*. In the first step, we show how to extract a subset of information from the complete metadata collection for visualization. In the second step, we compare numerous similar values available with in one metadata file and generate an overview sheet, eg. for addition into a lab book.

1.0.1 Download of published metadata

The published datasets are hosted on GIN and can be accessed directly via the webinterface or the local gin client. Here we download the metadata files of the two datasets directly.

[ ]:
```
# helper function by Ali Faki, https://stackoverflow.com/questions/7243750/
↪download-file-from-web-in-python-3
from requests import get
def download(url, file_name):
    # open in binary mode
    with open(file_name, "wb") as file:
        # get request
        response = get(url)
        # write to file
        file.write(response.content)

# location of the repository and metadata files on GIN
gin_repo = "https://web.gin.g-node.org/INT/multielectrode_grasp/raw/master/"
filenames = ["i140703-001.odml", "l101210-001.odml"]
filepaths = ["datasets/" + f for f in filenames]

# download metadata files from GIN
for filepath, filename in zip(filepaths, filenames):
    download(gin_repo + filepath, filename)
```

1.0.2 Extraction of a subset of metadata

The metadata collections of the electrophysiological experiment contain due to the complexity of the experiment thousands of entries organized in a hierarchical odML structure. This is of advantage for the storage of the metadata in an organized fashion, but for human interaction and visualization the amout of data needs to be reduced to a subset relevant for the current question. This can be achieved using the filter functionality of odMLtables. Here we extract all metadata related to the subject performing the task, which includes information such as the species, name, birthdate and handedness.

Since the publication of the datasets odML was developed further, so an updated odML version exists. In a first step we are converting the metadata files from odML file version 1.0 to file version 1.1 using the version conversion tool provided by odML. In a second step we use odMLtables to select only information related to the subject of the experiment and store this in a separate metadata file.

[ ]:
```
# inplace conversion of odML files to latest odML version
from odml.tools.version_converter import VersionConverter
for filename in filenames:
    converter = VersionConverter(filename)
    converter.convert()
    converter.write_to_file(filename)



# load odml file using odmltables and extract subset of information
import odmltables as odt

# extract only directly related subject information
for filename in filenames:
    odmlfile = odt.OdmlTable(filename)
    odmlfile.filter(SectionName='Subject')
    new_filename = filename.split('.')[0] + '_filtered.odml'
    odmlfile.write2odml(new_filename)
```

The generated odML files, which contain only a subset of the metadata can now be visualized in a browser using the odML style sheet. For this just place the style sheet in the same folder as your odML file and open the odML file in your browser. Here we use a helper function to display the odML content using the odML style sheet.

[ ]:
```
# This is utility code for displaying the odML file as html representation here.
# You can also just open the odML file in your browser having the style sheet␣
 ↪in the same location as your odML file and
# will get the same result
from IPython.display import display, HTML
import lxml.etree as ET

def display_odML_as_html(odML_file, xsl_file='odml.xsl'):
    # generate html representation from odML file and style sheet
    dom = ET.parse(odML_file)
    xslt = ET.parse(xsl_file)
    transform = ET.XSLT(xslt)
```
